# Validating Indicators of Disaster Recovery with Qualitative Research

**DOI:** 10.1371/currents.dis.ec60859ff436919e096d51ef7d50736f

**Published:** 2014-12-16

**Authors:** Caroline Dwyer, Jennifer Horney

**Affiliations:** Coastal Hazards Center, University of North Carolina at Chapel Hill, Chapil Hill, North Carolina, USA; Department of Epidemiology and Biostatistics, School of Public Health, Texas A&M Health Science Center, College Station, Texas, USA

## Abstract

Introduction: Recovery from disasters is a critical function of federal, state, and local governments, yet measurable, validated indicators of community recovery remain unidentified. A list of potential recovery indicators was developed by the authors through a literature review, recovery plan review, and case study of two disaster impacted communities. Methods: To validate the indicators, qualitative data was collected from experts on disaster recovery. Twenty-one key informant interviews and two focus groups were conducted between January and April of 2014 to solicit feedback from disaster recovery practitioners and academics. Results: Five major themes emerged from the qualitative data. These included: the flexibility of the indicators to serve multiple purposes for communities and individuals both pre- and post- disaster; the focus areas are comprehensive, but content and organization can be improved; the importance of seeing the indicators as a self-assessment, rather than a tool for comparing communities; the potential challenges of collecting data for some indicators; and the identification of potential measurement issues with the indicators. Discussion: The proposed recovery indicators can be utilized by both practitioners and researchers to effectively track post-disaster recovery. They capture many of the complexities of community disaster recovery and provide potential opportunities for linkages to the development of disaster recovery plans and other activities that could increase community resilience in the future.

## Introduction

Recovery from disasters is a critical function administered by federal, state, and local governments, yet measurable, validated indicators of community recovery remain unidentified. As part of an ongoing effort to develop a set of metrics for evaluating community recovery, 21 key informant interviews and two focus groups were conducted between January and April 2014 to solicit feedback from disaster recovery practitioners and academics. The metrics were derived from prior research conducted by a University of North Carolina Coastal Hazards Center (UNC-CHC) team. The research team initially identified 651 potential disaster recovery indicators through a detailed literature review, which were subsequently aggregated into 90 indicators by reviewing recovery plans and conducting case studies. The team further aggregated these indicators into 15 “recovery focus areas” and assigned primary and secondary metrics to each focus area. Interviews and focus groups were conducted to determine the feasibility of this proposed framework for the 15 recovery focus areas and their accompanying metrics; the main themes and issues raised by key informants are the focus of this report. This research will ultimately be used to develop a web-based tool to help communities track their recovery progress following a disaster.

## Background

Developing the Indicators

Over the last decade, the hazards research community has made the case for more systematic ways of measuring the disaster recovery process across events and over time.^1^^,^ 2 In the existing literature, a lack of common measurements and a primary focus on case studies of single disasters, limits the ability to compare outcomes between disasters as well as to monitor changes over time. To effectively improve community resilience in the face of disasters yet to come, there is a need for long-term coordinated research, with systematically collected and shared data.^3^^,^ 4 ^,^ 69 The development of a robust and user-friendly set of recovery indicators (i.e., markers used to designate a certain state or level), with associated quantifiable metrics (i.e., a means of measurement), can address many of these shortcomings, as well as support and build capacity of local practitioners by providing data for decision making during recovery. The proposed indicators would also assist in the development of a rigorous fact base, necessary for the development of a high-quality recovery plan during the recovery and redevelopment period, as well as assist in the development and implementation of a successful road map for the recovery process.^5^^,^ 6 ^,^ 7

A systematic review of the recovery literature, including 118 peer-reviewed sources and conference proceedings, revealed potential indicators that were catalogued and categorized by Federal Emergency Management Agency (FEMA) Recovery Support Function (RSF) or Recovery Mission Area Core Capability (Federal Emergency Management Agency, 2014). Fifty-seven of 118 (48.3%) of the publications and presentations included potential indicators of recovery. The 57 sources utilized various research methods including case studies, comparative case studies, longitudinal case studies, content analyses and literature reviews. They also represent multiple types of natural hazards, with hurricanes, tsunamis, and coastal storms being the most common (n=19), followed by earthquakes (n=10). The studies include multiple locations in the U.S. (n=38), as well as major international disasters (n=19) 8 ^-^ 64

Six hundred and fifty-one conceivable indicators were identified and categorized by a primary RSF or Core Capability based on the descriptions given in the National Disaster Recovery Framework or the National Preparedness Goal (Table 1) 65 ^,^ 66 . The RSFs and Core Capabilities were used because they provide an effective coordinating structure for the key functional areas of assistance typically needed by a community after a disaster. The 651 indicators were further consolidated via an inductive process to remove duplicates and provide a more succinct and user-friendly list of 90 indicators. All indicators were reviewed independently by two graduate student reviewers to identify duplicates or highly similar indicators as part of the process of reducing the number of total indicators from 651 to 90.

**Table 1. Indicators identified in literature review and pre-disaster recovery plan review, listed by Recovery Support Function (RSF)/Core Capability:** 

RSF/ Core Capability	Total Indicators (Literature Review)	Total Indicators (Recovery Plan Review)	Aggregated Indicators
Community Planning and Capacity Building	104	51	8
Economic Recovery	70	28	18
Health and Social Services Recovery	70	17	13
Housing Recovery	99	45	17
Infrastructure Systems Recovery	77	27	9
Natural and Cultural Resources Recovery	40	12	12
Public Information and Warning	19	4	6
Operational Coordination	43	20	7
**TOTAL**	**651**	**204**	**90**

Several methods were used to content validate this final, aggregated set of proposed indicators including: 1) a review of previously content-analyzed pre-disaster recovery (PDR) plans from 87 counties and municipalities located on the U.S. Atlantic and Gulf coasts; 2) a case study of two communities recently affected by disaster (Hoboken, New Jersey and New Hanover County, North Carolina); and 3) interviews with key informants and expert focus groups (the principal focus of this paper). Pre-disaster recovery plans were assessed identifying 204 indicators, which were then categorized by RSF or Core Capability. Each of these plan-based indicators could be categorized within one of the 90 already-proposed aggregate indicators, evidence of the plan review validating the existing literature-based indicators (see Table 1). The plan-based indicators were further reviewed to identify potential quantitative or qualitative evaluation metrics to be added to the list of possible indicator measurements.

A retrospective review of two case study communities undergoing recovery from recent disasters (Hoboken, New Jersey and New Hanover County North Carolina) was also conducted to content validate the indicators identified within the first phases of this project. For each community, researchers attempted to collect both the baseline (pre-event) and current status (as of July 2013) data needed to measure each of the proposed indicators. Pre-disaster, baseline conditions for the proposed 90 indicators were determined with relative ease for both communities with the largest amount of baseline data available for indicators categorized within the Economic Recovery and Housing Recovery RSFs and the Operational Coordination Core Capability. Many of the indicators also had current status data available; those most represented tended to be categorized within Economic Recovery, Housing Recovery, and Infrastructure Systems Recovery.

Following the first two phases of indicator content validation, the 90 aggregate indicators were further consolidated into a more useable form based on the PDR plan and case study validation processes. The aggregated indicators were organized into 15 focus areas (Table 2) and primary and secondary metrics were assigned to each focus area. These 15 focus areas and associated metrics were then used to solicit feedback from disaster recovery experts for the third phase of validation - the key informant interviews and focus groups.

**Table 2. Fifteen Proposed Recovery Focus Areas:** 

Business Recovery	Increased Participation in Disaster Planning	Repair or Replacement of Buildings and Infrastructure
Communication of Disaster-Related Information	Mobilization of Recovery Funding	Restoration of Cultural Sites/Resources
Economic Stabilization	Household Recovery	Restoration of Natural Resources
Healthy Communities	Population Stabilization	Social Services Recovery
Implementation of Hazard Mitigation Techniques	Public Sector Recovery	Use of Recovery Plan/Planning Measures

Key Informant Interviews and Focus Groups

The “complex, multi-dimensional, nonlinear” nature of disaster recovery demands a rigorous approach to any research attempting to better understand the ways in which we can assess a community’s recuperation following a disruptive event 67 . The described tendency of existing academic literature to focus on singular disaster events limits the weight of the indicators identified within published reports. One means to combat this shortcoming is to thoroughly vet the suggested indicators through a process of conducting key informant (KI) interviews and expert focus groups 68 . This method of synthesis research into the disaster recovery experience - combining the quantitative metrics obtained through the literature review phase with the largely qualitative data obtained through the KI interviews - is indicative of the multi-disciplinary future of disaster and recovery investigation 67 .

Twenty-one KI interviews and two focus groups were conducted during January and February 2014 to solicit feedback on the proposed focus areas and metrics developed for this project. The methods used to conduct these feedback sessions are outlined in subsequent sections, followed by the specific input provided by participants regarding both the overall project and the proposed focus areas and metrics of community recovery.

## Methods

Interview Guide

Interviews and guided discussions took a semi-structured format, with a number of close-ended questions asked of all participants, as well as unique follow-up questions dependent on the responses provided by the contributors. Questions included in the interview guide relate to both individual metrics as well as to the larger focus areas under which the metrics have been organized. Participants were asked to consider both the validity of the metric as well as the feasibility of collecting data to measure the characteristic in question.

Participants

The UNC-CHC team identified 28 potential interview participants with direct experience and knowledge, through either research or practice, of the proposed focus areas and potential metrics (Table 3). Potential interviewees were sent an email invitation to participate in a brief interview for the project. Twenty-two of the 28 participants (79%) replied to state their interest in the project and 21 (95%) participated in the interview. Additionally, two focus groups were conducted with a total of 10 expert participants to obtain additional feedback on the tool and metrics.

**Table 3. Summary of Interview and Focus Group Participants:** 

	ACADEMICS	PRIVATE PRACTITIONERS	PUBLIC PRACTITIONERS	TOTAL PARTICIPANTS
KI INTERVIEWS	4	2	15	21
FOCUS GROUP 1	1	1	3	5
FOCUS GROUP 2	3	1	1	5

Procedures

UNC research team members conducted all interviews either in-person or via telephone; focus groups were facilitated in-person. Each interview and focus group was recorded and transcribed. Transcripts were analyzed for key themes using inductive or open coding (i.e., themes are not predetermined, but rather emerge from data through examination and comparison. The project was reviewed and determined exempt by the University of North Carolina at Chapel Hill Institutional Review Board (#13-4017).

## Findings

Five major themes emerged from the key informant interviews and focus groups, pertaining to the potential development of an online Recovery Indicators Tool, the proposed focus areas, and the proposed metrics. This feedback is a critical component of the project, as it will inform the ongoing development of a web-based tool for community practitioners. Each theme, supported by specific participant responses is discussed, in detail, in the following section.

1. The Recovery Indicators Tool will potentially serve multiple purposes, both pre- and post-disaster

The majority of participants noted that a tool like the one currently under development is useful as a means to “get people thinking” about specific community elements that need to be addressed to both prepare for a potential disaster as well foster a successful recovery following a disaster event. One participant described the tool as a “focusing mechanism” that would keep “momentum moving forward” during recovery. Another respondent suggested the tool might function as an “executive playbook” for community decision-making in the aftermath of a disaster, particularly if decision-makers have not received training in emergency management. Other participants envisioned the tool being used for evaluations of the community, above and beyond the assessment of community recovery. Some ideas for potential uses include a “pre-disaster self-assessment” generated from baseline data entered into the tool or a means of measuring the “adaptive capacity” of a community in order to evaluate the ability to quickly recover from a disaster. The tool is meant to be flexible and the collection of pre- or inter-disaster data will be encouraged for comparison to post-disaster data, which may be collected and updated at various time points throughout recovery. Additionally, it was suggested that the tool could be used post-disaster to roughly estimate the level of recovery assistance needed by the community.

2. The proposed recovery Focus Areas are comprehensive but there is room for improvement in both their content and organization.

While it was noted that the proposed Focus Areas “align nicely” with the National Disaster Recovery Framework, many interviewees found redundancies between some of the Focus Areas (“Business Recovery” and “Economic Stabilization,” specifically) and suggested that combining similar areas might ease both organizational understanding and data collection/entry. There was some concern that the Focus Areas are not “intuitive” and “hard to digest” making it difficult to understand how the areas fit together during the disaster recovery experience. Similarly, another participant noted “separating metrics into Focus Areas makes it difficult to capture the interconnections that exist between different metrics and characteristics.” A suggestion was made to improve the proposed Focus Areas by creating thematic “clusters” to organize the Focus Areas in order to highlight similarities and interconnections. Finally, it was noted that the importance and/or relevance of specific Focus Areas might be “highly dependent” on the characteristics of the impacted community as well as the type of disaster experienced.

*3. Using metrics to measure or gauge recovery progress might lead to unfair comparisons between communities, or worse, the development of a “moral hazard” situation when disaster recovery is judged to be “complete” and the assistance-funding stream is terminated*.

Participant feedback noted that it is critical to clearly communicate the purpose of the Recovery Indicators Tool to communities, in order to assure users that the tool is a self-assessment and is not designed for inter-community comparative purposes. Interviewees suggest clearly articulating the “end goal” of the tool- a means to measure a community’s recovery progress- and soliciting the assistance of a local “project champion” to increase trust and community buy-in. Other suggestions to help overcome doubts are to develop a means of incentivizing use of the tool as well as to demonstrate the usefulness of the tool to the community by focusing on the “story” that can be told through the metrics and data collected. In addition to concerns about comparisons being made, one key informant noted there is the potential for a “moral hazard” to develop. This was described as a situation where a tool intended to help the community recover actually ends up causing harm due to a subjective determination being made, regarding the status of disaster recovery. This interviewee was troubled at the thought of recovery assistance streams being prematurely cut-off due as a result of using the Tool.

4. Potential data collection and reporting issues exist.

A number of participants noted potential issues that might exist relating to data collection and reporting. Local government units with less capacity may have difficulty dedicating time and/or staff to tracking the necessary baseline and post-disaster data that is required for the tool to serve its purpose. Respondents also mentioned the critical need for users to receive training in order to properly operate the tool and interpret the results, including how to collect useable, “good” data and how to interpret changes and trends in the collected data. One concern that remains challenging is the availability of easily accessible, open source data for all metrics.

Key informants made two suggestions to overcome potential data collection issues: 1) provide communities with likely data sources for individual metrics; and 2) pre-populate data within the Recovery Indicators Tool where possible in order to ease the burden on users. Participants noted it might also be necessary for users to acquire data from “non-traditional” sources (such as a local Chamber of Commerce) to fill any gaps in publically available data. Additionally, if data is not directly available for a metric, proxies might be required. An illustrative example provided by one respondent is a case in Colorado where a disaster-impacted community, heavily dependent on the natural environment, is using tourism revenue as a proxy to gauge the recovered value of natural resources. A final issue relating to data collection and interpretation raised during interviews is the importance of evaluating spatial data in addition to quantitative and qualitative data. Respondents expressed concern that the allocation of recovery funding is not always primarily driven by the degree of disaster impact. It was suggested that including spatial data, linking the damages and recovery assistance, in the Recovery Indicators Tool might improve assistance delivery and support greater accountability.

5. Potential issues with metrics and measurements exist.

A variety of suggestions and critiques of the metrics and measurements contained within the Focus Areas of the Recovery Indicators Tool were discussed. A frequent comment concerned the recovery timeframe being addressed through the metrics; interviewees had a difficult time interpreting whether the Tool was assessing short-term or long-term recovery outcomes. Some confusion also existed as to whether metrics were prioritized in some way- the primary, secondary and tertiary categorization of metrics within Focus Areas appeared to unnecessarily complicate the tool, based on interview responses. A number of participants expressed concern over “assigning quantitative indicators to fundamentally qualitative measures” (i.e. how a community “feels” in the recovery period). Other respondents suggested that some metrics seemed vague and that others might simply be “un-measurable” (one example provided was the metric relating to ecosystem resilience). A general comment on using metrics to gauge recovery was that the “focus of measurements should be outcomes not outputs, for example it is better to measure the number of schools re-opened rather than dollars spent on school recovery.” Supporting the case for the precedence of whole-system functionality over costs, another key informant discussing transportation systems expressed “cost doesn’t really capture what’s important… we need qualitative indicators of how well the system is working.” Two final metric-related issues raised are: 1) social equity must be addressed by identifying specific metrics relating to community equity; and 2) a community’s plan for post-disaster recovery and redevelopment will change the way metrics are interpreted and used. For instance, the data collected will be utilized quite differently if a “new normal is on the horizon” rather than if a community is “aiming to go back to what it was before the disaster.”

## Discussion

The process of validating the recovery indicators has revealed that utilization of a Recovery Indicators Tool can potentially serve as an effective means to track the recovery of a community or jurisdiction following a disaster. The robust qualitative feedback obtained through the KI interviews and focus groups supplements the context-limited data collected during the first phase of the research, allowing for the identification of indicators that more fully capture the complexity of community disaster recovery. The input of practitioners was particularly useful as these individuals are often the most intimately engaged in the practicalities of post-disaster community recovery, in a way that is not fully captured in the academic literature.

While the KI feedback helped to fill research gaps during the validation process, this methodology is not without limitations. The aspect in which this approach most often underperformed was the tendency of KIs to focus somewhat myopically (yet understandably) on their areas of specialization such as housing or the economy, often at the expense of other recovery focus areas. Additionally, researchers were often provided with suggestions for improvement of the tool, which were highly context- or disaster-specific and, therefore, not always relevant to the larger project. Overall, the use of the FEMA RSFs and Core Capabilities as an organizing framework may have inadvertently left out topics that are critical to a complete recovery, including mental health or overall quality of life. Potential gaps may need to be addressed with data collected via community surveys or other data collection tools. Finally, as the tool under developed is intended for use by practitioners, no input was gathered from community members impacted by disaster, who may offer different perspectives on priorities and measurement of progress during recovery.

## Conclusions

Overall, participants responded in a positive manner to both the concept of the Recovery Indicators Tool as well as its content. Following the completion of the key informant feedback phase of this project, the research team has amended the Focus Areas and accompanying metrics in order to incorporate the comments received, including combining redundant Focus Areas and metrics, organizing the Focus Areas within thematic groupings for purposes of clarity, and designating metrics relating to equity considerations within a recovering community (Figure 1). This updated version of the metrics will be used in the next phase of the project: pilot testing the web-based Recovery Indicators Tool in a selection of communities impacted by Superstorm Sandy in 2012. While it is likely that this Tool will undergo further refinements in the future, even this initial iteration helps to fill a critical need for greater accountability and transparency during the process of community recovery following a disaster.

**Revised Focus Area and Metrics:**
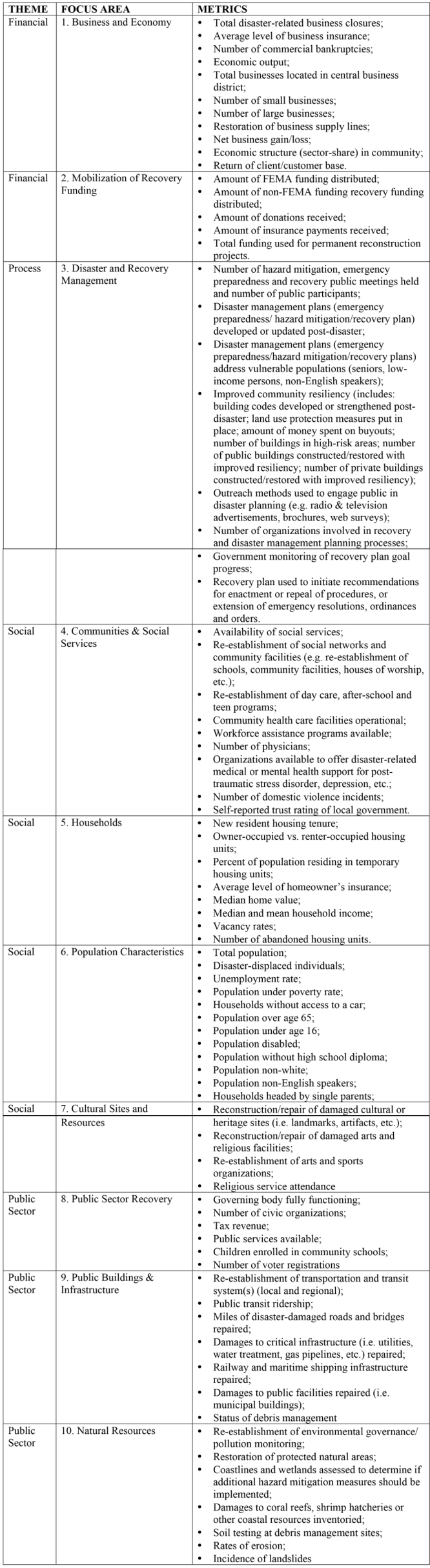

## Competing Interests

The authors have declared that no competing interests exist.
